# Evaluation of OCT image quality in gas-filled eyes using four OCT devices

**DOI:** 10.1186/s40942-026-00879-2

**Published:** 2026-06-05

**Authors:** Mitsuru Otsubo, Tomoko Ro-Mase, Shun Konno, Ami Konno, Harumasa Yokota, Taiji Nagaoka

**Affiliations:** Department of Ophthalmology, Asahikawa Medical University, 2-1-1-1 Midorigaoka Higashi, Asahikawa, Hokkaido 078-8510 Japan

**Keywords:** Optical coherence tomography, Gas tamponade, Macular hole, Image quality, Postoperative imaging.

## Abstract

**Purpose:**

To compare optical coherence tomography (OCT) image quality obtained on postoperative day 1 under intraocular gas tamponade using four OCT devices, including two swept-source OCT (SS-OCT) and two spectral-domain OCT (SD-OCT) systems.

**Methods:**

This retrospective study included 30 consecutive eyes of 30 patients who underwent pars plana vitrectomy with gas tamponade between June 2025 and February 2026. On postoperative day 1, OCT imaging was performed using four devices: two SS-OCT systems (DRI OCT Triton and PLEX Elite 9000) and two SD-OCT systems (RTVue XR Avanti and SPECTRALIS OCT). OCT image quality was graded using a 5-point scale by two masked graders, and the mean score was used for analysis. The primary analysis compared image quality scores among devices in 25 eyes with macular hole using the Friedman test with Bonferroni correction.

**Results:**

The mean patient age was 70.0 ± 8.7 years, and 13 patients (43.3%) were male. The median (interquartile range) image quality scores in macular hole cases (*n* = 25) were 5.0 (4.0–5.0) for Triton, 5.0 (4.0–5.0) for Plex Elite, 4.0 (4.0–5.0) for Avanti, and 2.0 (1.0–3.0) for Spectralis. Image quality scores differed significantly among the four devices (Friedman test, *p* < 0.001).

**Conclusions:**

Under intraocular gas tamponade, the SD-OCT device Avanti demonstrated relatively preserved image quality. These findings suggest that device-specific acquisition characteristics, beyond wavelength differences, substantially influence OCT image quality in gas-filled eyes. These findings may assist clinicians in selecting appropriate OCT devices for reliable postoperative assessment under gas tamponade.

## Introduction

Gas tamponade is a widely used surgical technique in vitreoretinal surgery for conditions such as rhegmatogenous retinal detachment and macular hole. However, ophthalmoscopic visualization is often impaired in gas-filled eyes, making detailed postoperative intraocular assessment challenging. Optical coherence tomography (OCT) has been shown to be useful for early postoperative evaluation of the macula under gas tamponade, and its utility has been reported since the era of Fourier-domain OCT [1, 2]. 

With technological advances, spectral-domain OCT (SD-OCT) and swept-source OCT (SS-OCT) have enabled relatively improved imaging even under gas-filled conditions. OCT-based assessment of the macula under gas tamponade has also been used to guide postoperative management, including adjustment of facedown positioning duration in macular hole surgery [1–6]. Nevertheless, OCT imaging in gas-filled eyes remains technically challenging because image quality is often degraded by reflections at the gas–fluid interface and by myopic shifts in the focal plane [7]. 

SS-OCT has been reported to provide better image quality than SD-OCT owing to its longer imaging wavelength under gas-filled conditions [6, 8–10]. However, direct comparisons of OCT image quality between SD-OCT and SS-OCT under gas tamponade are limited. To date, only one study by Ahn et al. (2018) has compared OCT images obtained under gas-filled conditions. That study evaluated earlier-generation devices, specifically the 3D-OCT 2000 (SD-OCT) and the DRI-OCT (SS-OCT) (Topcon Corporation, Tokyo, Japan) [10]. To our knowledge, no study has systematically compared OCT image quality under gas tamponade across multiple contemporary OCT devices.

Therefore, the purpose of this study was to compare OCT image quality obtained on postoperative day 1 under intraocular gas tamponade using four different OCT devices, including two SS-OCT and two SD-OCT systems.

## Methods

### Study design and participants

This retrospective study included 30 consecutive eyes of 30 patients who underwent pars plana vitrectomy with gas tamponade between June 2025 and February 2026. All eyes underwent OCT imaging with four devices—two SS-OCT systems and two SD-OCT systems—on postoperative day 1. The analysis included eyes that were pseudophakic and had a clear anterior segment, with no hyphema, fibrin formation, or corneal opacity, at the time of OCT imaging on postoperative day 1. Eyes with clinically significant corneal edema associated with Descemet folds or impaired foveal visualization, as well as eyes with obviously insufficient intraocular gas fill, were excluded from the analysis. This restriction was applied to ensure a uniform optical environment and to minimize confounding factors affecting OCT image quality under gas tamponade. Data were collected retrospectively from electronic medical records. The study was conducted in accordance with the tenets of the Declaration of Helsinki and was approved by the institutional review board of Asahikawa Medical University (approval number: 24072). Informed consent was obtained using an opt-out method.

### Demographic and clinical data

Collected demographic and clinical data included age, sex, primary diagnosis, surgical procedure, tamponade material, intraocular pressure, axial length, postoperative spherical equivalent (diopters), and postoperative lens status.

### Surgical procedure

Vitrectomy was performed using the Constellation Vision System (Alcon, Fort Worth, TX, USA). A 25-gauge or 27-gauge system was used, and either three-port or four-port vitrectomy was performed by four experienced surgeons (M.O., A.K., H.Y., T.N.). Internal limiting membrane visualization was assisted using 0.025% Brilliant Blue G dye when necessary according to the surgical condition. Gas tamponade was achieved using either air or sulfur hexafluoride (SF_6_), selected at the surgeon’s discretion. When SF6 was used, a single injection of 0.8–1.5 mL was administered to achieve an intraocular concentration of approximately 20%. Patients with rhegmatogenous retinal detachment were instructed to maintain a face-down position for 1 day postoperatively, whereas patients with macular hole were instructed to maintain a face-down position for 1–2 days postoperatively.

### Ophthalmic examinations and OCT image acquisition

Comprehensive ophthalmic examinations, including best-corrected visual acuity, intraocular pressure measurement, slit-lamp biomicroscopy, and indirect ophthalmoscopy, were performed preoperatively and postoperatively in all patients.

On postoperative day 1, OCT imaging was performed using four devices: DRI OCT Triton (Topcon Corporation, Tokyo, Japan; Triton), PLEX Elite 9000 (Carl Zeiss Meditec, Dublin, CA, USA; Plex Elite), RTVue XR Avanti (Optovue, Fremont, CA, USA; Avanti), and SPECTRALIS OCT (Heidelberg Engineering, Heidelberg, Germany; Spectralis).

Triton and Plex Elite are SS-OCT devices with a central wavelength of 1050 nm and a scan speed of 100,000 A-scans/s. Avanti is an SD-OCT device with a central wavelength of 840 nm and a scan speed of 70,000 A-scans/s, while Spectralis has a central wavelength of 870 nm and a scan speed of 40,000 A-scans/s. Axial resolution was 8 μm for Triton, 6 μm for Plex Elite, 5 μm for Avanti, and 7 μm for Spectralis. The dioptric focus ranges were − 33 to + 40 D for Triton when using the built-in compensation lens, − 20 to + 20 D for Plex Elite, − 15 to + 20 D for Avanti, and − 12 to + 12 D for Spectralis. These specifications were obtained from the manufacturers’ technical documentation.

All images were acquired using line scans: 9-mm line scans for Triton and Plex Elite, 12-mm line scans for Avanti, and 30° (approximately 9 mm) line scans for Spectralis. Frame averaging was used to improve OCT image quality. In Spectralis imaging, the automatic real-time (ART) mode was used, in which multiple B-scan frames acquired at the same retinal location are averaged to generate a single image. The number of averaged frames was as follows: Triton (16 frames), Plex Elite (100 frames), Avanti (60 frames), and Spectralis (ART 30).

All OCT examinations were performed by the same experienced examiner (T.M.). Imaging was performed under gas-filled conditions with careful adjustment of focus. When tracking was active, two scans were obtained and the better image passing through the fovea was selected. When tracking was inactive, two scans were similarly obtained and the better image was selected. Because Spectralis requires active tracking to complete image acquisition, scans were considered unsuccessful when tracking failed. All OCT examinations were performed under pharmacological mydriasis.

### Image quality assessment

**Fig. 1:**
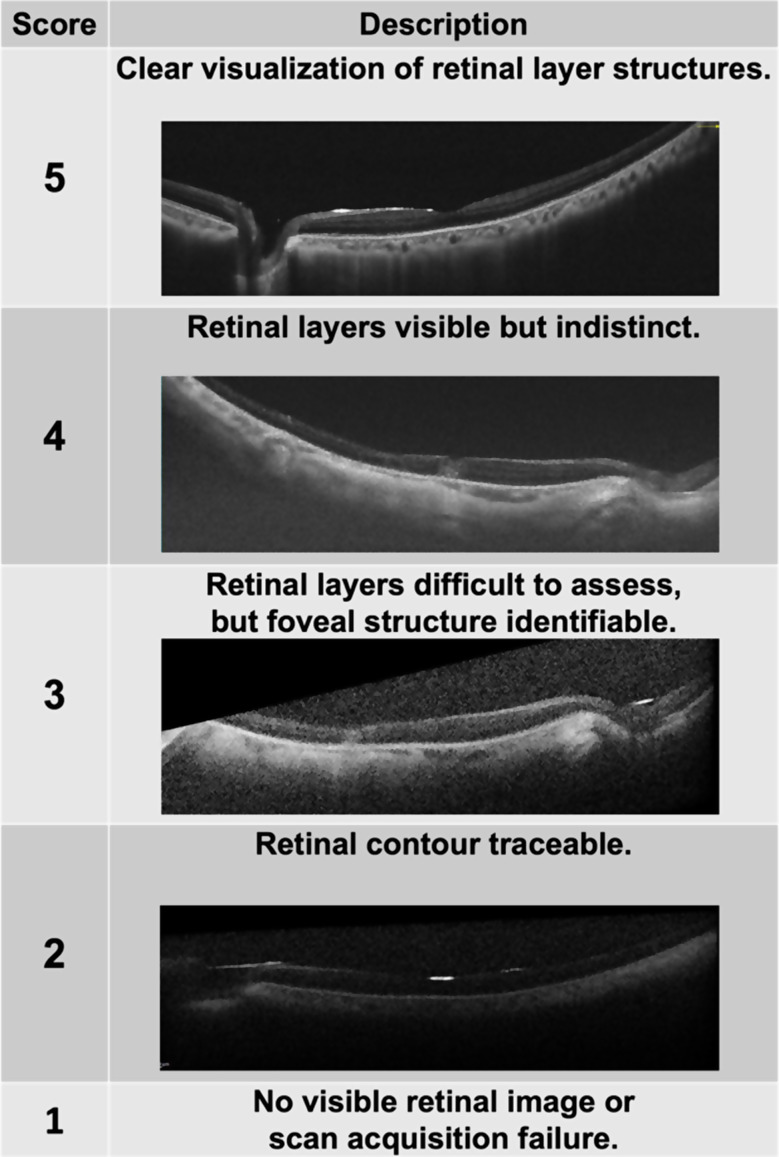
Representative OCT images corresponding to each image quality score. Representative OCT images obtained from actual study cases illustrating the five-point image quality scoring system used in this study. Scores range from 5 (clear visualization of retinal layer structures) to 1 (no visible retinal image or scan acquisition failure)

OCT images obtained on postoperative day 1 under gas tamponade were graded using a 5-point scale: 5, clear visualization of retinal layer structures; 4, retinal layers visible but indistinct; 3, retinal layers difficult to assess, but foveal structure identifiable; 2, retinal contour traceable; 1, no visible retinal image or scan acquisition failure. Images were independently evaluated by two masked graders, and the mean score was used as the image quality score. Representative OCT images illustrating each image quality score are shown in Fig. 1.

### Statistical analysis

Statistical analyses were performed using EZR (Saitama Medical Center, Jichi Medical University, Saitama, Japan), a graphical user interface for R (The R Foundation for Statistical Computing, Vienna, Austria). For the primary analysis, which was limited to macular hole eyes to allow consistent evaluation of foveal structure across devices, OCT image quality scores were compared among the four devices using the Friedman test with Bonferroni correction for post hoc comparisons. Additional exploratory analysis restricted to successfully acquired Spectralis images was performed to separately evaluate image quality independent of acquisition failure related to mandatory active tracking.

Secondary analyses included assessment of feasibility (defined as an image quality score ≥ 3), tracking completion rates, and inter-grader agreement. Feasibility and tracking completion rates were evaluated using descriptive statistics. Inter-grader agreement was assessed using weighted Cohen’s κ.

Because Spectralis OCT has a limited dioptric focus range and requires successful tracking for image acquisition, postoperative spherical equivalent was compared between eyes with successful and unsuccessful tracking using the Mann–Whitney U test. Reasons for scan failure were classified as unknown when no specific cause could be identified. Statistical significance was set at *p* < 0.05.

## Results

A total of 30 eyes from 30 patients were included in the analysis. The mean age was 70.0 ± 8.7 years, and 17 patients (56.7%) were male. The primary diagnosis was macular hole in 25 eyes (83.3%), while other diagnoses included lamellar macular hole (2 eyes, 6.7%), rhegmatogenous retinal detachment (2 eyes, 6.7%), and vitreomacular traction syndrome (1 eye, 3.3%). Vitrectomy alone was performed in 6 eyes (20.0%), and combined vitrectomy with phacoemulsification and intraocular lens implantation was performed in 24 eyes (80.0%). SF₆ gas was used as the tamponade material in 27 eyes (90.0%), while air was used in 3 eyes (10.0%). The mean intraocular pressure was 15.0 ± 2.7 mmHg. The mean axial length was 24.89 ± 1.94 mm. The mean postoperative spherical equivalent was − 2.16 ± 2.16 D. Postoperatively, 30 eyes (100.0%) were pseudophakic (Table 1).

**Table 1:** Characteristics of the subjects included in the analysis

Characteristic	Overall (*n* = 30 eyes)
Age, years	70.0 ± 8.7
Sex, n (%)	
Male	17 (56.7)
Female	13 (43.3)
Primary diagnosis, n (%)	
Macular hole	25 (83.3)
Other diseases	LMH, 2 (6.7); RRD, 2 (6.7); VMTS, 1 (3.3)
Surgical procedure, n (%)	
PPV	6 (20.0)
PPV + PEA+IOL	24 (80.0)
Tamponade material, n (%)	
SF6	27 (90.0)
Air	3 (10.0)
Intraocular pressure, mmHg	15.0 ± 2.7
Axial length, mm	24.89 ± 1.94
Postoperative spherical equivalent, D	-2.16 ± 2.16
Postoperative lens status, n (%)	
Intraocular lens	30 (100.0)

Data are presented as mean ± standard deviation or number (%), as appropriate

LMH Lamellar macular hole, RRD Rhegmatogenous retinal detachment, VMTS Vitreomacular traction syndrome, PPV Pars plana vitrectomy, PEA+IOL Phacoemulsification and aspiration and intraocular lens implantation, SF6 Sulfahexafluoride

**Fig. 2:**
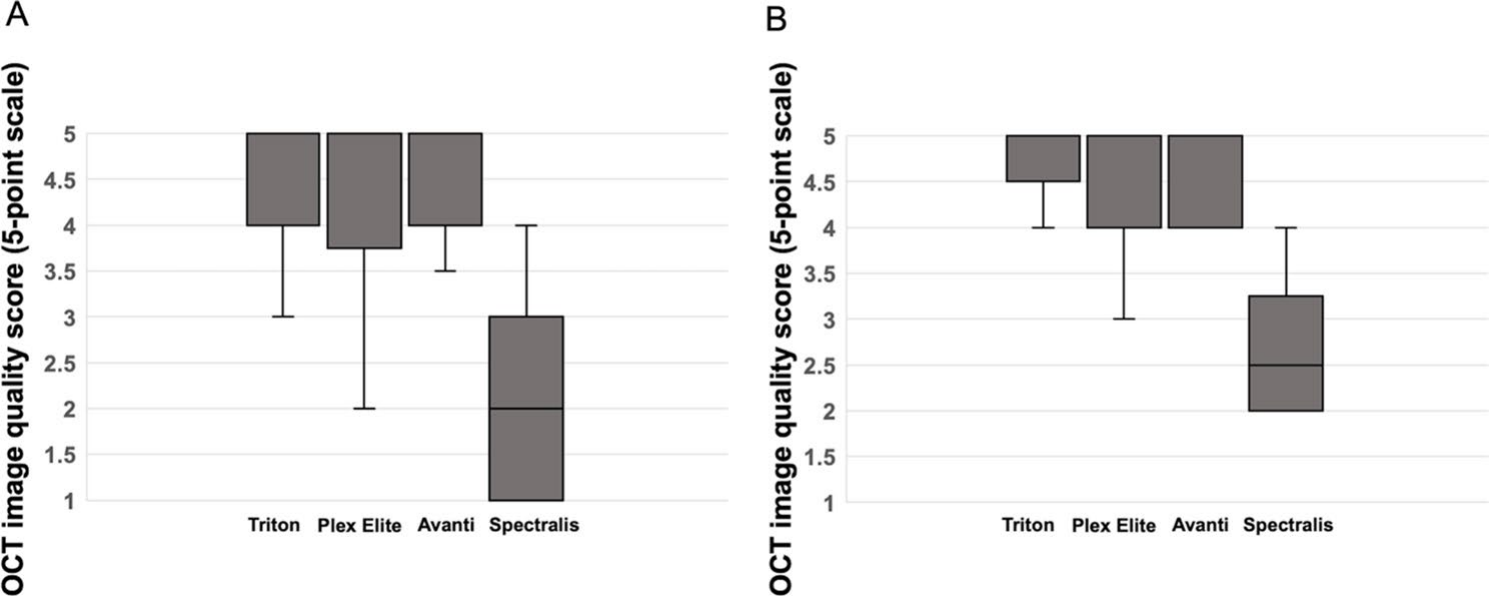
Box-and-whisker plots of OCT image quality scores under intraocular gas tamponade in eyes with macular hole. (**A**) Primary analysis including all macular hole eyes. (**B**) Exploratory analysis restricted to successfully acquired Spectralis images. Boxes represent the interquartile range (IQR), the horizontal line within each box indicates the median, and whiskers extend to 1.5×IQR. Individual data points represent each eye. OCT image quality was graded on a 5-point scale, with higher scores indicating better image quality. Image quality scores differed significantly among devices in both analyses (Friedman test, both *p* < 0.001)

**Fig. 3:**
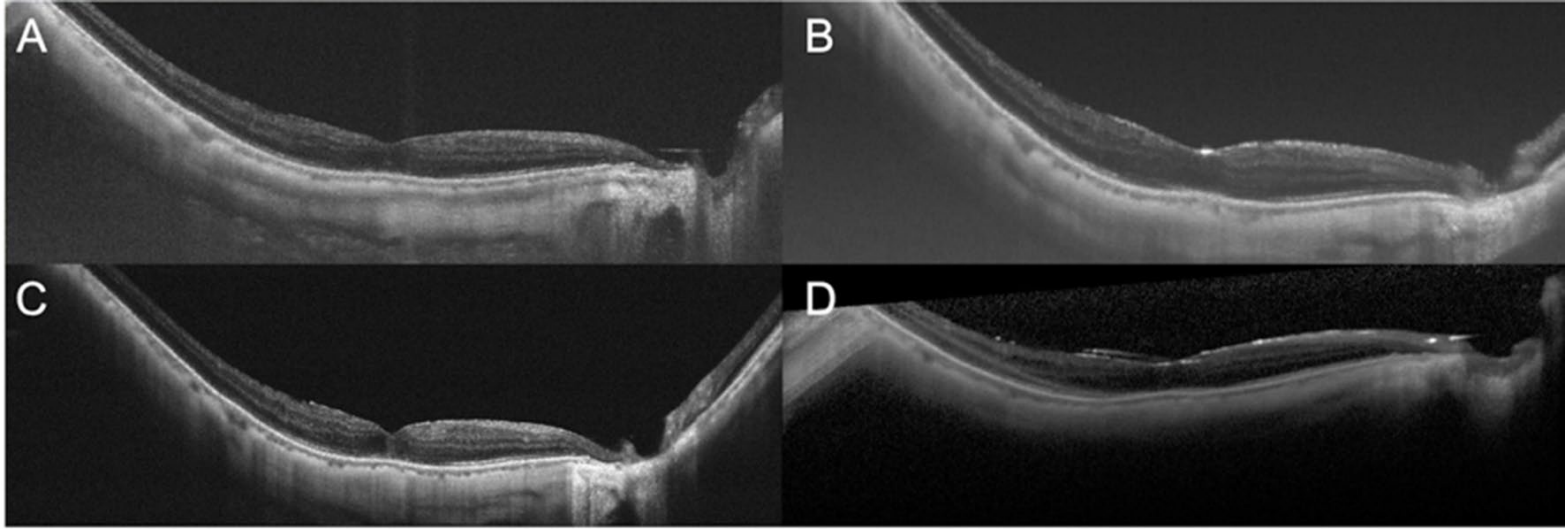
Representative OCT images obtained under intraocular gas tamponade in a macular hole eye on postoperative day 1. **(A)** DRI OCT Triton (Topcon Corporation, Tokyo, Japan). **(B)** PLEX Elite 9000 (Carl Zeiss Meditec, Dublin, CA, USA). **(C)** RTVue XR Avanti (Optovue, Fremont, CA, USA). **(D)** SPECTRALIS OCT (Heidelberg Engineering, Heidelberg, Germany). All images were acquired using horizontal line scans passing through the fovea in the same eye with a macular hole under intraocular gas tamponade. Differences in image quality among devices were observed, with clearer visualization of retinal layers in Triton, Plex Elite, and Avanti compared with Spectralis

At the time of OCT imaging, the gas meniscus extended beyond the inferior vascular arcade in all eyes, indicating that a sufficient intraocular gas fill was present in all cases. As the primary analysis, OCT image quality scores were compared among the four devices in 25 eyes with macular hole (Fig. 2A). The median (interquartile range) image quality scores were 5.0 (4.0–5.0) for Triton, 5.0 (4.0–5.0) for Plex Elite, 4.0 (4.0–5.0) for Avanti, and 2.0 (1.0–3.0) for Spectralis. Image quality scores differed significantly among the four devices (Friedman test, *p* < 0.001). Post hoc analysis revealed that Triton, Plex Elite, and Avanti showed significantly higher image quality scores than Spectralis (Bonferroni correction: Triton vs. Spectralis, *p* < 0.001; Plex Elite vs. Spectralis, *p* < 0.001; Avanti vs. Spectralis, *p* < 0.001). In an additional exploratory analysis restricted to successfully acquired Spectralis images in 18 eyes (Fig. 2B), the median (interquartile range) image quality scores were 5.0 (5.0–5.0) for Triton, 5.0 (4.0–5.0) for Plex Elite, 5.0 (4.0–5.0) for Avanti, and 2.5 (2.0–3.0) for Spectralis. Image quality scores differed significantly among the four devices (Friedman test, *p* < 0.001). Post hoc analysis revealed that Triton, Plex Elite, and Avanti showed significantly higher image quality scores than Spectralis (Bonferroni correction: Triton vs. Spectralis, *p* = 0.002; Plex Elite vs. Spectralis, *p* = 0.004; Avanti vs. Spectralis, *p* = 0.002). Representative OCT images obtained with each device under intraocular gas tamponade are shown in Fig. 3.

As secondary analyses, feasibility (defined as an image quality score ≥ 3), tracking completion rate, and inter-grader agreement were evaluated for all eyes (Table 2). Feasibility rates were 100.0% for Triton, 90.0% for Plex Elite, 100.0% for Avanti, and 26.7% for Spectralis. Tracking completion rates were 63.3%, 30.0%, 86.7%, and 63.3%, respectively. In many cases with unsuccessful image acquisition, it was difficult to identify a specific cause other than tracking failure, and the reasons for failure were therefore classified as unknown. Inter-grader agreement was high across all devices, with weighted Cohen’s κ values ranging from 0.74 to 0.96.

**Table 2:** Feasibility, tracking completion, and inter-grader agreement of OCT imaging under intraocular gas tamponade

Device	Feasibility *n* (%)	Tracking completion *n* (%)	Weighted κ
Triton	100.0	63.3	0.89
Plex Elite	90.0	30.0	0.96
Avanti	100.0	86.7	0.74
Spectralis	26.7	63.3	0.94

Weighted κ indicates inter-grader agreement between the two masked graders

In Spectralis OCT, postoperative spherical equivalent did not differ significantly between eyes with successful and unsuccessful tracking (median [IQR], −1.63 [−3.44 to −0.38] vs −1.25 [−3.50 to −0.44]; Mann–Whitney U test, p = 0.931).

## Discussion

In this study, we compared OCT image quality obtained under intraocular gas tamponade using four contemporary OCT devices, including two SS-OCT systems and two spectral-domain SD-OCT systems. Our results demonstrated significant differences in image quality among the devices on postoperative day 1. While the SD-OCT system Avanti demonstraited relatively preserved image quality, the SD-OCT system Spectralis showed significantly inferior performance compared with the other three devices.

Previous studies have generally suggested that SS-OCT provides superior image quality in gas-filled eyes due to its longer imaging wavelength, which allows better tissue penetration and reduced sensitivity to media opacity [8–11]. Indeed, previous studies have reported visualization rates of approximately 77.5–80.0% for SS-OCT and 76.2–92.3% for SD-OCT under gas tamponade [3, 7–9, 11, 12]. Ahn et al. reported that on postoperative day 1, high-quality OCT images were obtained in 88.9% of macular hole eyes using SS-OCT, whereas the success rate was only 59.3% with SD-OCT [10]. However, it should be noted that the SD-OCT device used in that study was an earlier-generation model (3D-OCT 2000, Topcon Corporation, Tokyo, Japan released in 2009), which may partly explain the relatively low imaging success rate.

In contrast, the SD-OCT device Avanti used in the present study achieved a high feasibility rate of 100.0%, similar to those observed with the two SS-OCT systems. This finding suggests that improvements in SD-OCT hardware, including faster scan speed, enhanced signal processing, and optimized acquisition algorithms, may mitigate some of the limitations traditionally associated with shorter imaging wavelengths. Therefore, OCT image quality under gas tamponade may not be determined solely by wavelength characteristics. Differences in frame averaging presets among devices may also have influenced subjective image quality assessment by affecting image noise reduction and retinal layer visibility. Interestingly, despite using the highest frame averaging settings among the evaluated devices, Plex Elite showed image quality scores comparable to those of Triton and Avanti, suggesting that factors other than frame averaging alone may also contribute to image quality under gas tamponade conditions.

In our analysis, Spectralis OCT showed a markedly lower feasibility rate (26.7%) compared with the other devices. Spectralis requires active tracking for image acquisition and has a relatively narrow dioptric focus range. We therefore compared postoperative spherical equivalent between eyes with successful and unsuccessful tracking; however, no significant difference was observed. This suggests that refractive status alone does not fully explain the high rate of tracking failure. Other factors, such as specular reflection at the gas–fluid interface, optical distortion due to refraction at the interface, and device-specific tracking algorithms, may play a more substantial role in determining image acquisition success under gas tamponade [7, 11]. 

Previous studies have reported that OCT imaging in gas-filled eyes is influenced by several factors, including myopic shifts of the focal plane, gas bubble volume, and strong reflections at the gas–fluid interface [7, 11]. Sano et al. reported that OCT imaging becomes difficult when the gas fill is ≤ 60% [3]. In the present study, the gas meniscus extended beyond the inferior vascular arcade in all cases at the time of OCT imaging, suggesting a relatively large gas fill. Therefore, insufficient gas volume was unlikely to have influenced the differences in image quality observed among the devices. Our findings support and extend these observations by demonstrating that OCT image quality under gas tamponade is governed by a complex interaction of optical factors and device-specific characteristics, rather than by imaging wavelength alone.

OCT imaging under gas tamponade is clinically important, particularly in macular hole surgery, where early confirmation of hole closure can guide postoperative management, including the duration of facedown positioning [1, 2, 4–6]. Although recent studies have reported successful macular hole closure without strict facedown positioning [9, 13, 14], a recent individual participant data meta-analysis demonstrated that facedown positioning was associated with a trend toward higher closure rates and better postoperative visual acuity, with statistically significant benefits observed in macular holes larger than 400 μm [15]. In this context, reliable OCT imaging under gas tamponade allows early assessment of macular hole closure, which may facilitate individualized postoperative positioning strategies, improve prognostic counseling, and reduce psychological burden on patients. These findings suggest that OCT imaging under gas tamponade can be reliably achieved with appropriate device selection, which may have important implications for postoperative management in clinical practice.

This study has several limitations. First, the analysis was restricted to pseudophakic eyes with a clear anterior segment to ensure a uniform optical environment; therefore, generalizability may be limited. Second, its retrospective design and relatively small sample size may limit the generalizability of our findings. Third, differences in dioptric focus range, tracking requirements, and scan protocols among devices could not be fully standardized. Although Triton is equipped with a built-in compensation lens that extends its myopic focus range, similar optical aids were not applied to other devices, as the present study was designed to evaluate image quality using commercially available systems in their standard real-world clinical configurations. Objective quantitative metrics such as signal-to-noise ratio were not evaluated in the present study. In addition, standardized comparison of such parameters across different OCT platforms may be challenging because image processing and noise reduction algorithms differ among devices. No obvious qualitative differences in OCT image appearance were observed between air-filled and SF6-filled eyes in the present study, although the number of air-filled eyes was limited.

Future studies with larger sample sizes and controlled imaging conditions are warranted to evaluate the diagnostic performance of OCT devices and to clarify the relative contributions of optical and algorithmic factors to image quality under gas tamponade.

In conclusion, our results indicate that Avanti SD-OCT demonstrated relatively preserved image quality under intraocular gas tamponade. These findings suggest that OCT image quality in gas-filled eyes cannot be explained solely by wavelength differences and highlight the importance of device-specific optical design and acquisition algorithms. These findings may assist clinicians in selecting appropriate OCT devices for reliable postoperative assessment under gas tamponade.

## Data Availability

The datasets generated and/or analyzed during the current study are available from the corresponding author on reasonable request.

## References

[1] Eckardt C, Eckert T, Eckardt UTE, Porkert UTE, Gesser C. Macular Hole Surgery with Air Tamponade and Optical Coherence Tomography-Based Duration of Face-down Positioning. Retina. 2008;28(8):1087–96. 18779715 10.1097/IAE.0b013e318185fb5f

[2] Masuyama K, Yamakiri K, Arimura N, Sonoda Y, Doi N, Sakamoto T. Posturing time after macular hole surgery modified by optical coherence tomography images: a pilot study. Am J Ophthalmol. 2009;147(3):481–e488482. 19061988 10.1016/j.ajo.2008.09.028

[3] Sano M, Inoue M, Taniuchi S, Kunita D, Hiraoka T, Hirakata A. Ability to determine postoperative status of macular hole in gas-filled eyes by spectral-domain optical coherence tomography. Clin Exp Ophthalmol. 2011;39(9):885–92. 21631685 10.1111/j.1442-9071.2011.02586.x

[4] Shah SP, Manjunath V, Rogers AH, Baumal CR, Reichel E, Duker JS. Optical Coherence Tomography–Guided Facedown Positioning for Macular Hole Surgery. Retina. 2013;33(2):356–62. 23343822 10.1097/IAE.0b013e318263d0e8 PMC3758113

[5] Yamashita T, Sakamoto T, Yamashita T, Sonoda S, Yamakiri K, Otsuka H, Hisatomi T, Imaki H, Ishibashi T, Dugel PU. Individualized, Spectral Domain-Optical Coherence Tomography–Guided Facedown Posturing after Macular Hole Surgery. Retina. 2014;34(7):1367–75. 24955569 10.1097/IAE.0000000000000087

[6] Sato M, Iwase T. Swept source-optical coherence tomography-guided facedown posturing to minimize treatment burden and maximize outcome after macular hole surgery. J Clin Med. 2023;12(16). 10.3390/jcm12165282 PMC10455272 37629324

[7] Goto K, Mizukawa K, Kiryu J. Factors affecting imaging of spectral-domain optical coherence tomography in gas-filled eyes after macular-hole surgery. Jpn J Ophthalmol. 2012;56(3):236–44. 22350383 10.1007/s10384-012-0122-y

[8] Li DQ, Choudhry N. Swept-Source OCT Visualization of Macular Hole Closure in Gas-Filled Eyes. Ophthalmic Surg Lasers Imaging Retina. 2017;48(5):392–8. 28499050 10.3928/23258160-20170428-05

[9] Zhang Y, Chen X, Hong L, Yan Y, Zeng M, Huang Z, Liu R, Ding Q. Facedown Positioning after Vitrectomy Will Not Facilitate Macular Hole Closure Based on Swept-Source Optical Coherence Tomography Imaging in Gas-Filled Eyes. Retina. 2019;39(12):2353–9. 30204729 10.1097/IAE.0000000000002325

[10] Ahn SJ, Park SH, Lee BR. Visualization of the Macula in Gas-Filled Eyes. Retina. 2018;38(3):480–9. 28196050 10.1097/IAE.0000000000001560

[11] Kikushima W, Imai A, Toriyama Y, Hirano T, Murata T, Ishibashi T. Dynamics of macular hole closure in gas-filled eyes within 24 h of surgery observed with swept source optical coherence tomography. Ophthalmic Res. 2015;53(1):48–54. 25531151 10.1159/000368437

[12] Yamashita T, Yamashita T, Kawano H, Sonoda Y, Yamakiri K, Sakamoto T. Early imaging of macular hole closure: a diagnostic technique and its quality for gas-filled eyes with spectral domain optical coherence tomography. Ophthalmologica. 2013;229(1):43–9. 23095234 10.1159/000343061

[13] Nadal J, Delas B, Piñero A. Vitrectomy without face-down posturing for idiopathic macular holes. Retina. 2012;32(5):918–21. 22080914 10.1097/IAE.0b013e318229b20e

[14] Alberti M, la Cour M. NONSUPINE POSITIONING IN MACULAR HOLE SURGERY: A Noninferiority Randomized Clinical Trial. Retina. 2016;36(11):2072–9. 27046458 10.1097/IAE.0000000000001041

[15] Raimondi R, Tzoumas N, Toh S, Sarohia GS, Phillips MR, Chaudhary V, Steel DH. Facedown Positioning in Macular Hole Surgery: A Systematic Review and Individual Participant Data Meta-Analysis. Ophthalmology. 2025;132(2):194–205. 39147105 10.1016/j.ophtha.2024.08.012

